# Glycine Suppresses AGE/RAGE Signaling Pathway and Subsequent Oxidative Stress by Restoring Glo1 Function in the Aorta of Diabetic Rats and in HUVECs

**DOI:** 10.1155/2019/4628962

**Published:** 2019-03-03

**Authors:** Ziwei Wang, Junqing Zhang, Lei Chen, Jingjing Li, Hong Zhang, Xiaohui Guo

**Affiliations:** Department of Endocrinology, Peking University First Hospital, No. 8 Xishiku Avenue, Xicheng District, Beijing 100034, China

## Abstract

Oxidative stress plays a crucial role in the pathogenesis of diabetic vascular complications. It is known that the accumulation of advanced glycation end products (AGEs) and the activation of the receptor of AGEs (RAGE) induce sustained oxidative stress in the vascular tissue. Growing evidence indicates that glycine, the simplest amino acid, exerts antioxidant and antiglycation effects and also improves vascular function. However, the mechanism whereby glycine protects vascular tissue against oxidative stress in models with diabetes has not been investigated. In the present study, we evaluated whether glycine can attenuate oxidative stress by suppressing the AGE/RAGE signaling pathway in the aorta of streptozotocin-induced diabetic rats and in human umbilical vascular endothelial cells (HUVECs). Our results showed that oral glycine administration increased NO content and ameliorated oxidative stress in the serum and aorta of diabetic rats. The AGE/RAGE signaling pathway in the aorta of diabetic rats was significantly attenuated by glycine treatment as manifested by decreases in levels of AGEs, RAGE, Nox4, and NF-*κ*B p65. The suppressive effect of glycine on the formation of AGEs was associated with increased activity and expression of aortic glyoxalase-1 (Glo1), a crucial enzyme that degrades methylglyoxal (MG), the major precursor of AGEs. In MG-treated HUVECs, glycine restored the function of Glo1, suppressed the AGE/RAGE signaling pathway, and inhibited the generation of reactive oxygen species. In addition, the reduction in the formation of AGEs in HUVECs caused by glycine treatment was inhibited by Glo1 inhibition. Taken together, our study provides evidence that glycine might inhibit the AGE/RAGE pathway and subsequent oxidative stress by improving Glo1 function, thus protecting against diabetic macrovascular complications.

## 1. Introduction

Vascular complications have become the leading cause of morbidity and mortality among patients suffering from diabetes mellitus worldwide. Oxidative stress plays a central role in the pathogenesis of diabetic vascular complications [1]. It is well established that the formation of advanced glycation end products (AGEs) and the subsequent signaling pathway contribute in a major way to the sustained oxidative stress that occurs in the vascular tissue [2, 3]. AGEs are formed by nonenzymatic reactions between reducing sugars and amino groups of large biomolecules, including proteins, nucleic acids, and lipids [4]. This irreversible process is accelerated under chronic hyperglycemia and/or oxidative stress, as occurs with diabetes mellitus. In addition to being deposited in the extracellular matrix and recruiting macrophages in the vessels [5], AGEs also bind to the receptor of AGEs (RAGE) and activate NADPH oxidase (Nox) and NF-*κ*B [6], thus initiating a vicious cycle of oxidative stress and inflammation [7, 8]. Another deleterious feature of AGEs is their role in “metabolic memory.” Since the formation of AGEs cannot be reversed, their accumulation in the vascular tissue induces sustained oxidative stress, even if hyperglycemia is improved [9]. Therefore, finding ways to inhibit AGE formation is of particular importance to protect against oxidative stress in diabetic vascular injury.

Methylglyoxal (MG), a highly reactive dicarbonyl metabolite of glycolysis, has been increasingly recognized as the major precursor of intracellular AGEs [10]. MG is degraded by the glyoxalase system, an efficient enzymatic detoxification system, of which glyoxalase-1 (Glo1) is the rate-limiting enzyme [11]. With glutathione (GSH) as a cofactor, Glo1 converts MG into an intermediate product, which is further detoxified into lactate by glyoxalase-2. Under diabetic conditions, both Glo1 expression and GSH levels are decreased [12–14]. Therefore, the function of Glo1 is impaired, leading to uncontrolled AGE formation and oxidative stress [15, 16]. Hence, an enhancement of Glo1 function would play a valuable role in inhibiting this detrimental process.

Glycine is the simplest amino acid in mammals. Besides participating in synthesizing structural biomolecules, glycine serves as one of the predecessors of GSH, one of the most important antioxidants in the human body. In diabetic complications, glycine exerts suppressive effects on glycation, such as delaying cataract formation [17], although the mechanism for this has not been clearly established. Recent studies have reported some protective effects of glycine on vascular injuries, such as improving endothelial function [18] and restoring vascular reactivity [13], but the effects of glycine on large blood vessels exposed to diabetic conditions have not been investigated. The antiglycation and antioxidant effects of glycine leave open the possibility that glycine might work by suppressing AGE formation and inhibiting the activation of the AGE/RAGE axis, thus protecting against oxidative stress and diabetic vascular complications. Due to the effect of glycine in restoring vascular GSH levels [13], we speculated that glycine may exert beneficial effects on Glo1 function, thus restoring the ability of Glo1 to inhibit AGE formation.

In the present study, we aimed to investigate the effect of glycine on the AGE/RAGE signaling pathway as well as on the function of Glo1 in the aorta of diabetic rats and in human umbilical vascular endothelial cells (HUVECs). Our goal was to increase the number of possible therapeutic methods that may be useful against diabetic macrovascular complications.

## 2. Materials and Methods

### 2.1. Experimental Animals

All protocols and procedures of our study were approved by the Ethics Committee for Animal Experimentation of the Faculty of Peking University First Hospital (approval number: J201613).

Six-week-old male Sprague-Dawley (SD) rats were housed under a 12 h light-dark cycle. All animals had unlimited access to drinking water and a chow diet. After two weeks of acclimation, all rats were randomly assigned either to an experimental group or to a healthy control group. After an overnight fast, the experimental group was intraperitoneally injected with a single dose of streptozotocin (STZ, 45 mg/kg of bodyweight, Sigma-Aldrich, St. Louis, MO, USA). Diabetes mellitus (DM) was confirmed 2 weeks later by measuring blood glucose levels (individuals with levels > 16.7 mmol/L were confirmed as diabetic). The experimental group was further divided into a DM group (nontreated diabetic rats receiving normal drinking water, *N* = 8) and a DG group (diabetic rats receiving 1% (*w*/*v*) glycine *ad libitum* in drinking water [13, 19], *N* = 8). Likewise, the healthy control group was divided into two groups: a control group (healthy rats receiving normal drinking water, *N* = 7) and a CG group (healthy rats receiving 1% (*w*/*v*) glycine *ad libitum* in drinking water, *N* = 7). After 12 weeks, all rats were anesthetized with pentobarbital and sacrificed. The thoracic aortas were quickly removed and stored at -80°C or immersed in formalin for fixation. Serum samples were collected to determine the biochemical profile (by Automatic Biochemical Analyzer 7600, Hitachi, Tokyo, Japan) and glycine concentration.

### 2.2. Measurement of Serum Glycine

Serum samples were prepared using the EZ:faast GCMS Free Amino Acid Analysis Kit (Phenomenex, Torrance, CA, USA) and analyzed on a Gas Chromatograph-Mass Spectrometer-QP2010 (Shimadzu, Kyoto, Japan) according to the manufacturer's instructions. The conditions were as follows: samples were injected using split injection at a ratio of 1 : 10 and a port temperature of 280°C. An Rtx-5MS column (30 m × 0.25 mm) was used to separate the compounds. The initial oven temperature was set at 100°C, then raised to 300°C at a rate of 10°C/min, and then held for 10 minutes.

### 2.3. Histopathology

The descending thoracic aortas were fixed in 10% formalin, embedded in paraffin, and cut into 4 *μ*m sections. The rehydrated sections were stained with hematoxylin-eosin (H&E) to observe the overall changes and to measure the intima-media thickness (IMT). Five random nonoverlapping visual fields of one section were captured on an Olympus DP71 microscope (magnification 400x). In each visual field, the IMT was measured at four different points by ImageJ software. A Verhoeff-Van Gieson staining kit (DC0059, Leagene, Beijing, China) was used to observe the elastic fibers in the tissue.

### 2.4. Measurement of NO Concentration and Oxidative Stress Markers in the Serum and Aorta

The samples of the aorta were sonicated in ice-cold RIPA lysis buffer (P0013, Beyotime, Shanghai, China) and centrifuged at 16,000 g for 20 min at 4°C. The protein concentrations were determined by the Pierce BCA Protein Assay Kit (Thermo Fisher Scientific, Hudson, NH, USA) and adjusted to the same levels. Equal amounts of soluble aorta homogenates and serum samples were assayed to determine levels of NO metabolites (S0021, Beyotime, Shanghai, China), total GSH levels (S0053, Beyotime, Shanghai, China), malondialdehyde (MDA) levels (S0131, Beyotime, Shanghai, China), and superoxide dismutase (SOD) activity (706002, Cayman Chemical, Ann Arbor, Michigan, USA), following the manufacturers' instructions.

### 2.5. Measurement of AGE Levels

100 *μ*l of the solubilized protein samples was pipetted into the wells of a 96-well nontransparent plate, and the autofluorescence of AGEs was assessed at Ex 370 nm/Em 440 nm wavelengths [20]. The AGE ELISA kit (MBS261131, MyBioSource, San Diego, CA, USA) was also applied to determine the concentrations of AGEs, following the manufacturer's instructions. The data were normalized to protein concentrations.

### 2.6. Immunohistology

We followed the methods of Wang et al. [21]. After blocking, the aorta sections were incubated overnight at 4°C with a rabbit anti-AGE antibody (1 : 200, ab23722, Abcam, Cambridge, UK), rabbit anti-RAGE antibody (1 : 200, ab3611, Abcam, Cambridge, UK), rabbit anti-Nox4 antibody (1 : 150, ab133303, Abcam, Cambridge, UK), mouse anti-3-nitrotyrosine antibody (1 : 200, ab61392, Abcam, Cambridge, UK), rabbit anti-NF-*κ*B p65 antibody (1 : 200, D14E12, CST, Beverly, MA, USA), or mouse anti-Glo1 antibody (1 : 200, MA1-13029, Invitrogen, Waltham, MA, USA). The sections were washed and then incubated with a secondary antibody (1 : 200, peroxidase-conjugated anti-rabbit (ZB-2301) or anti-mouse (ZB-2305) antibody, ZSGB-BIO, Beijing, China) for 1 hour. Color was developed using DAB. Images were captured using an Olympus DP71 microscope. The mean IOD of staining (IOD/area) from 5 random fields on one section was assessed using Image-Pro Plus 6.0 software.

### 2.7. Western Blot

The solubilized protein samples were boiled with loading buffer for 5 min. Equal amounts of protein (30 *μ*g) were separated using 12% SDS-polyacrylamide gel electrophoresis and electrotransferred onto a nitrocellulose membrane. The membrane was blocked with 5% skim milk and incubated with a rabbit anti-RAGE antibody (1 : 1000, ab3611, Abcam, Cambridge, UK), rabbit anti-Nox4 antibody (1 : 1000, ab133303, Abcam, Cambridge, UK), rabbit anti-NF-*κ*B p65 antibody (1 : 1000, D14E12, CST, Beverly, MA, USA), or mouse anti-Glo1 antibody (1 : 3000, MA1-13029, Invitrogen, Waltham, MA, USA) overnight at 4°C. After a thorough washing, the membrane was incubated with a secondary antibody (1 : 5000, peroxidase-conjugated anti-rabbit (ZB-2301) or anti-mouse (ZB-2305) antibody, ZSGB-BIO, Beijing, China) for an hour at room temperature. *β*-Actin was used as the loading control (1 : 3000 TA-09, ZSGB-BIO, Beijing, China). The bands were visualized using ECL Western Blotting Substrate (Thermo Fisher Scientific, Hudson, NH, USA) and quantified by Image-Pro Plus 6.0 software.

### 2.8. Measurement of Glo1 Activity

The activity of Glo1 was measured according to the method established by Arai et al. [22]. The samples were washed in PBS and sonicated in 10 mM sodium phosphate buffer (pH = 7). After centrifugation, the protein concentration was determined by the BCA kit and adjusted to the same level in all samples. In a 96-well plate, 125 *μ*l sodium phosphate buffer (100 mM, pH = 6.6), 25 *μ*l GSH (40 mM), 25 *μ*l MG (40 mM), and 70 *μ*l Millipore water were added into each well and incubated at 37°C for 15 min. After incubation, 5 *μ*l solubilized protein from cells or from aortic tissue was added into each well. The absorbance at 240 nm (*A*_240_) was monitored every 2 min. The Glo1 activity was displayed as the rate of change in *A*_240_ per mg protein.

### 2.9. Cell Culture and Viability

The human umbilical vein endothelial cell line (HUVEC) was bought from the Chinese National Infrastructure of Cell Line Resource. The HUVECs were cultured in minimum essential media (MEM without glycine, glucose 5.5 mM, Hyclone, Life Science, Pittsburgh, PA, USA) with 10% fetal bovine serum (Gibco, Life Science, Pittsburgh, PA, USA), 100 U/ml penicillin, and 100 *μ*g/ml streptomycin at 37°C in a humidified atmosphere of 5% CO_2_. The HUVECs were identified by their typical cobblestone morphology and the presence of von Willebrand factor (VWF) antigen.

Cell viability was evaluated using CCK-8 (Cell Counting Kit-8, Donjindo, Mashikimachi, Japan). 3 × 10^3^ HUVECs were seeded into each well of a 96-well plate. After treatment, each well was washed gently with phosphate-buffered saline (PBS) to exclude possible interference from any remaining culture medium. 10 *μ*l CCK-8 solution and 100 *μ*l new MEM media without phenol red were added to each well. After incubation at 37°C for 3 hours, the absorbance at 450 nm was measured.

### 2.10. Immunofluorescence

HUVECs were seeded onto a cover glass placed in a well of a 12-well plate. After treatment, all pieces of cover glass were incubated in 10% neutral formalin for 10 min, washed 3 times with PBS, and incubated with 0.1% Triton X-100 reagent for 10 min. The cells were then blocked in 1% BSA for 30 min and incubated with 50 *μ*l rabbit anti-AGE antibody (1 : 200, ab23722, Abcam, Cambridge, UK) overnight at 4°C. After extensive washing in PBS, the cells were incubated in a fluorescent secondary antibody for an hour in a dark chamber. After washing 3 times, the cover glass was mounted with DAPI. Digital images of fluorescent staining were captured on the Olympus microscope. Five random nonoverlapping visual fields of one section were captured to calculate the mean fluorescence intensity.

### 2.11. Estimation of Intracellular Reactive Oxygen Species (ROS)

Intracellular ROS was estimated by flow cytometry using an oxidation-sensitive fluorescent probe (2′,7′-dichlorofluorescin diacetate, DCFH-DA, D6883, Sigma-Aldrich, St. Louis, MO, USA). The treated cells were gently washed with PBS and incubated with DCFH-DA (10 *μ*M in MEM without phenol red). After 30 minutes, the cells were washed with PBS 3 times and digested by 0.05% trypsin-EDTA. The mean fluorescence intensities at Ex 488/Em 525 were detected by flow cytometry.

### 2.12. Statistical Analysis

The statistical analysis of the data was performed using SPSS 20.0 (SPSS Inc., Chicago, USA) following the methods published elsewhere [21]. Quantitative data are presented as the means ± standard errors of the means (for normally distributed data) or medians and interquartile ranges (for nonnormally distributed data). Differences between groups were assessed using a one-way analysis of variance (ANOVA) for normally distributed data, followed by Tukey's post hoc test. In addition, the Kruskal-Wallis test was used for nonnormally distributed data. A *p* value less than 0.05 was considered significant.

## 3. Results

### 3.1. Effect of Glycine on Plasma Glucose, Body Weight, and Serum Glycine Levels

At week 12, the plasma glucose levels in the DM group were significantly higher than those in the control group (*p* < 0.001, Table 1). The body weight and serum glycine levels in the DM group were lower than those in the control group (*p* < 0.001 and *p* < 0.05, respectively). Compared with the DM group, the glucose levels and body weight in the DG group seemed unaffected, whereas the serum glycine levels were significantly increased (*p* < 0.001).

### 3.2. Effects of Glycine Treatment on Aortic Histopathology and Vascular Function

**Figure 1:**
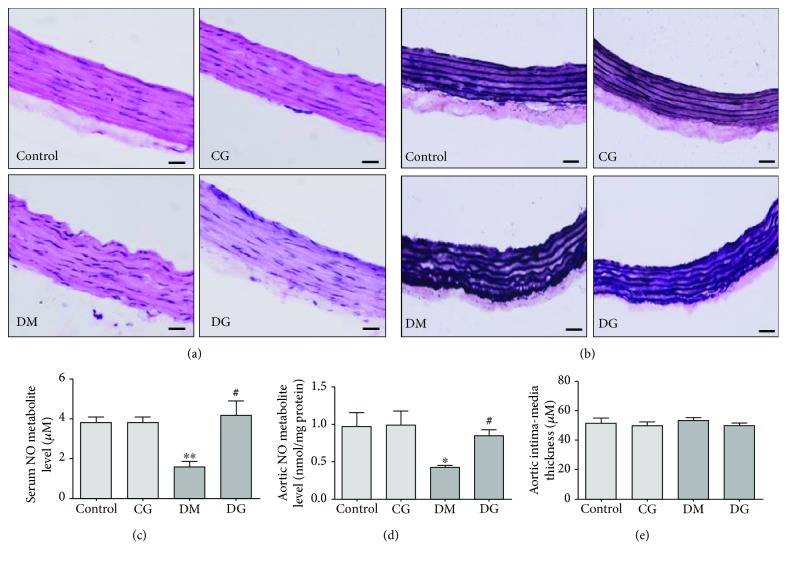
Alterations in aorta histopathology and NO concentrations in the four groups. (a) Representative images of HE staining in sections of the rat aorta, magnification 400x. (b) Representative images of Verhoeff-Van Gieson staining in sections of the rat aorta, magnification 400x. The scale bar indicates 20 *μ*m. (c) Serum NO metabolite levels after 12 weeks of treatment. *N* = 6. (d) NO metabolite levels in the rat aorta after 12 weeks of treatment. *N* = 6. (e) The aortic intima-media thickness after 12 weeks of treatment. *N* = 7-8. Control: healthy rats receiving normal tap water. CG: healthy rats receiving water containing 1% (*w*/*v*) glycine. DM: diabetic rats receiving normal tap water. DG: diabetic rats receiving water containing 1% (*w*/*v*) glycine. ^∗^*p* < 0.05, ^∗∗^*p* < 0.01 compared with the control group; ^#^*p* < 0.05 compared with the DM group.

To evaluate the effect of glycine treatment on the structure of aortic tissue, we applied H&E and Verhoeff-Van Gieson staining to examine morphological changes. The H&E staining (Figure 1(a)) showed that in the DM group, both the intimal and medial layers of the aorta were disorganized, whereas much less injury was observed in the DG group. The Verhoeff-Van Gieson staining (Figure 1(b)) demonstrated that the elastic fibers in the DM group displayed severe fragmentation and distortion. With glycine treatment, the distortion of the elastin fibers was lower than that of the DM group, although not completely restored.

To assess the effect of glycine on endothelial vascular function, we measured the levels of NO metabolites in the serum and aorta. Figures 1(c) and 1(d) show that in the DM group, the concentrations of NO metabolites were significantly decreased both in the serum and in the homogenates of aortic tissue as compared with the control group (*p* < 0.01 and *p* < 0.05, respectively). In the DG group, the concentrations of NO metabolites were significantly elevated compared with these concentrations in the DM group (*p* < 0.05). As shown in Figure 1(e), no significant difference in aorta IMT was observed among the four groups.

### 3.3. Glycine Increases Antioxidant Capacity in the Serum of Diabetic Rats

**Figure 2:**
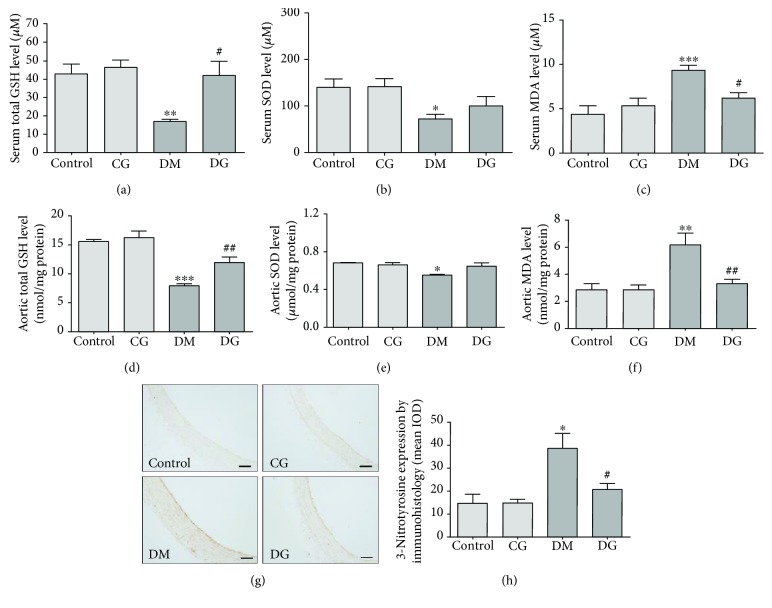
Oxidative markers in the serum and aorta homogenates. Total GSH levels (a), SOD levels (b), and MDA levels (c) in serum samples from different groups were measured after 12 weeks of treatment (*N* = 6). Total GSH levels (d), SOD levels (e), and MDA levels (f) in aorta homogenates from different groups were measured after 12 weeks of treatment (*N* = 6). (g) and (h) show the immunohistological analysis of 3-nitrotyrosine in the aorta, magnification 200x. The scale bar indicates 40 *μ*m. IOD: integrated optical density. *N* = 6. ^∗^*p* < 0.05, ^∗∗^*p* < 0.01, ^∗∗∗^*p* < 0.001 compared with the control group. ^#^*p* < 0.05, ^##^*p* < 0.01 compared with the DM group.

To assess the effect of glycine on antioxidant capacity, we measured the levels of GSH, SOD, and MDA in the serum and aorta of rats. In the serum, both GSH and SOD levels in the DM group were significantly decreased as compared with the control group (*p* < 0.01 and *p* < 0.05, respectively, Figures 2(a) and 2(b)). However, the GSH levels were significantly increased in the DG group as compared with the DM group (*p* < 0.05). The SOD levels in the DG group tended to be elevated, but this trend did not reach statistical significance. The serum MDA levels in the DM group were increased as compared with those in the control group (*p* < 0.001, Figure 2(c)), but this increase was abolished in the DG group (*p* < 0.05). No changes in these markers were observed in the CG group.

### 3.4. Glycine Restores Antioxidant Status in the Aorta of Diabetic Rats

In the aortic tissue homogenates, diabetes significantly decreased the GSH and SOD levels as compared with the control group (*p* < 0.001 and *p* < 0.01, respectively, Figures 2(d) and 2(e)), whereas glycine treatment significantly increased these two antioxidant markers (*p* < 0.01 and *p* < 0.05, respectively). The MDA levels in the aorta were increased in the DM group as compared with the control group (*p* < 0.01). With glycine treatment, the MDA levels in the diabetic rats were decreased (*p* < 0.01, Figure 2(f)). No obvious alterations were detected in the CG group as compared with the control group. As shown by immunohistology (Figures 2(g) and 2(h)), the expression of 3-nitrotyrosine in the aorta was significantly increased in the DM group as compared with the control group (*p* < 0.05), especially in the intimal layer. However, the DG group showed much less staining of 3-nitrotyrosine (*p* < 0.05).

### 3.5. Glycine Suppresses AGE Accumulation and the RAGE-Nox-NF-*κ*B Signaling Pathway

**Figure 3:**
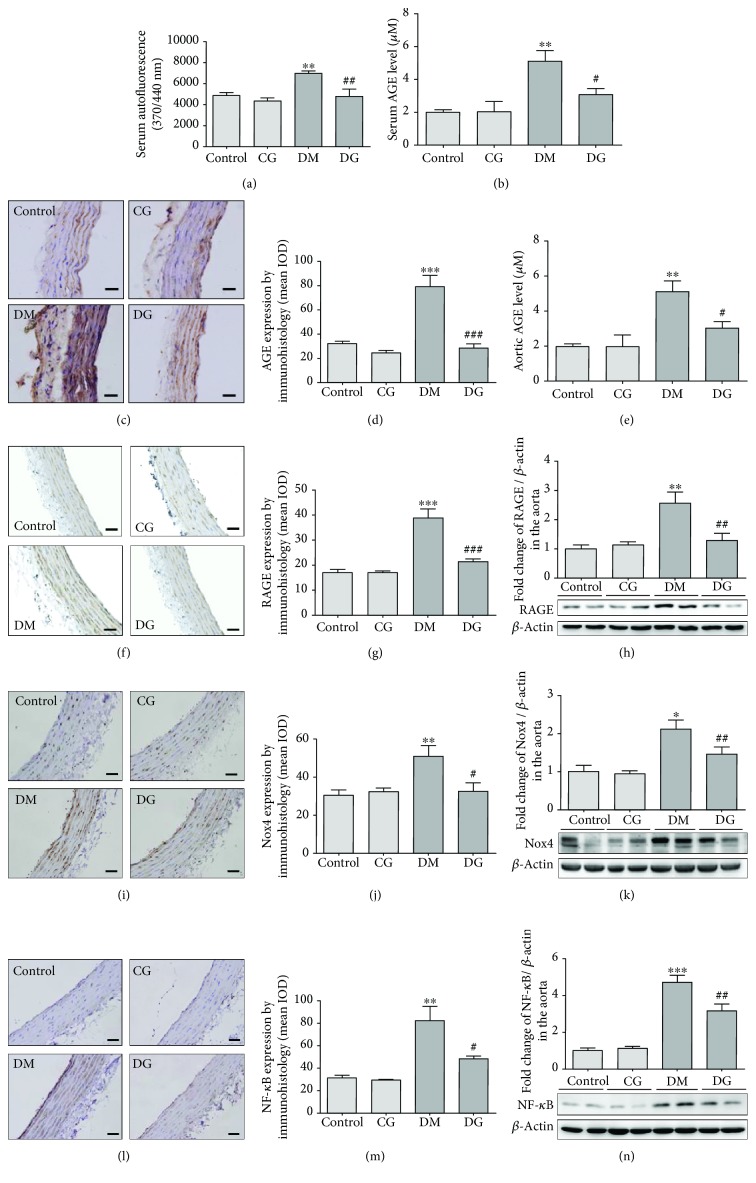
Analyses of AGEs, RAGE, Nox4, and NF-*κ*B p65 in the aorta from different groups after 12 weeks of treatment. (a) Serum AGE levels from different groups after 12 weeks of treatment were measured by autofluorescence at Ex 370 nm/Em 440 nm. *N* = 6. (b) Serum AGEs measured by ELISA. *N* = 6. (c) and (d) show the immunohistological analysis of AGEs in the aorta, magnification 400x. The scale bar indicates 20 *μ*m. *N* = 6. (e) AGE levels in the rat aorta measured by ELISA. *N* = 6. (f) and (g) show the immunohistological analysis of RAGE in the aorta, magnification 400x. The scale bar indicates 20 *μ*m. *N* = 6. (h) The expression of RAGE in the rat aorta determined by western blot. *N* = 6. (i) and (j) show the immunohistological analysis of Nox4 in the aorta, magnification 400x. The scale bar indicates 20 *μ*m. *N* = 6. (k) The expression of Nox4 in the rat aorta determined by western blot. *N* = 6. (l) and (m) show the immunohistological analysis of NF-*κ*B p65 in the aorta, magnification 400x. The scale bar indicates 20 *μ*m. *N* = 6. (n) The expression of NF-*κ*B p65 in the rat aorta determined by western blot. *N* = 6. ^∗^*p* < 0.05, ^∗∗^*p* < 0.01, ^∗∗∗^*p* < 0.001 compared with the control group. ^#^*p* < 0.05, ^##^*p* < 0.01, ^###^*p* < 0.001 compared with the DM group.

It is well known that AGE binds to its receptor RAGE, which further activates NADPH oxidase (Nox) [23] and inflammatory response [24], eventually causing a vicious loop of oxidative stress in the vascular tissue [2, 5]. Thus, the AGE/RAGE signaling pathway constitutes one of the major mechanisms of vascular oxidative stress. It was reported that glycine supplementation could reduce the level of AGEs in the serum of diabetic rats [17], but its effect in the vascular tissue stays unknown. To investigate whether the antioxidative effect of glycine was associated with suppression of the AGE/RAGE pathway, we measured the expression of AGEs, RAGE, Nox4, and NF-*κ*B p65. Serum AGE levels in the DM group were significantly increased in contrast with those in the control group (*p* < 0.01, Figures 3(a) and 3(b)). Glycine treatment reduced the AGE levels in serum as detected by both autofluorescence and ELISA (*p* < 0.01 and *p* < 0.05, respectively). Likewise, the aortic AGE levels were increased in the DM group as detected by immunohistological analysis (*p* < 0.001, Figures 3(c) and 3(d)) but were reduced in the DG group (*p* < 0.001). Similar results were also found when measuring aortic AGEs by ELISA (Figure 3(e)).

To investigate whether the glycine-induced decrease in AGEs could affect the downstream AGE/RAGE signaling pathway, we applied immunohistology and immunoblots to determine the expression of RAGE, Nox4, and NF-*κ*B p65 in the aorta. As shown in Figures 3(f)–3(n), the expressions of aortic RAGE, Nox4, and NF-*κ*B p65 increased significantly in the DM group as compared with the control group (*p* < 0.01, *p* < 0.05, and *p* < 0.001, respectively) in western blot analysis. However, the expressions of RAGE, Nox4, and NF-*κ*B in the DG group were significantly decreased as compared with those in the DM group (*p* < 0.01, *p* < 0.05, and *p* < 0.01, respectively). Similar results were also observed in the immunohistological analyses. No significant difference was noticed in the CG group as compared with the control group.

### 3.6. Glycine Increases Aortic Glo1 Activity and Protein Expression

**Figure 4:**
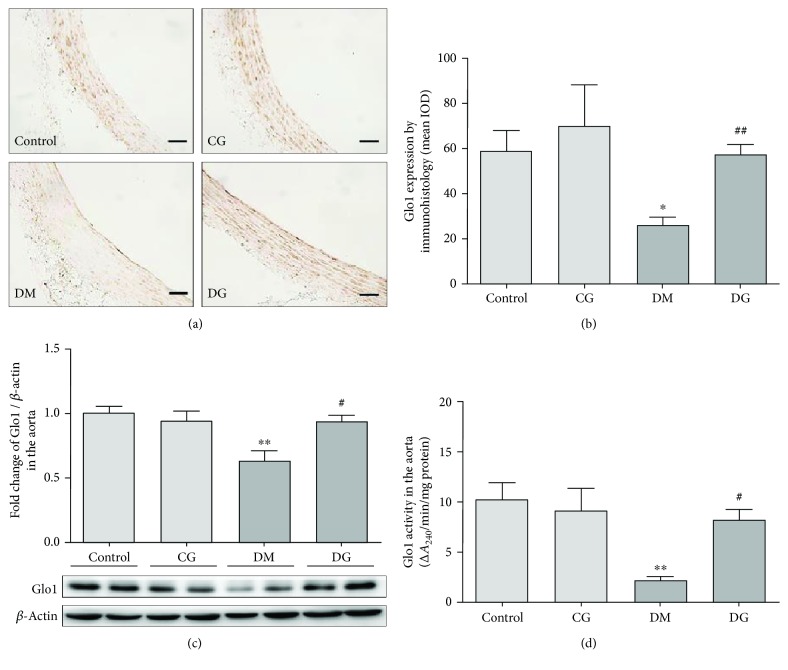
Immunohistological and immunoblot analyses of Glo1 and Glo1 activity measurements in the aorta from different groups after 12 weeks of treatment. (a) and (b) show the immunohistological analysis of Glo1 in the aorta, magnification 400x. The scale bar indicates 20 *μ*m. *N* = 6. (c) The expression of Glo1 in the rat aorta determined by western blot. *N* = 6. (d) The activity of Glo1 in the rat aorta. *N* = 6. ^∗^*p* < 0.05, ^∗∗^*p* < 0.01 compared with the control group. ^#^*p* < 0.05, ^##^*p* < 0.01 compared with the DM group.

To find out if the suppressive effect of glycine on AGE formation was due to the activation of the Glo1 system, we measured the activity and the protein expression of aortic Glo1. As shown in the immunohistological analysis (Figures 4(a) and 4(b)), much less expression of Glo1 was observed in the DM group than in the control group (*p* < 0.05), whereas a greater level of staining of Glo1 was shown in the DG group than in the DM group (*p* < 0.01). As shown in Figures 4(c) and 4(d), the expression and activity of aortic Glo1 were significantly lower in the DM group than in the control group (*p* < 0.01). However, both Glo1 protein expression and activity were markedly increased in the DG group as compared with the DM group (*p* < 0.05).

### 3.7. Glycine Restores Viability and Glo1 Function in MG-Treated HUVECs

**Figure 5:**
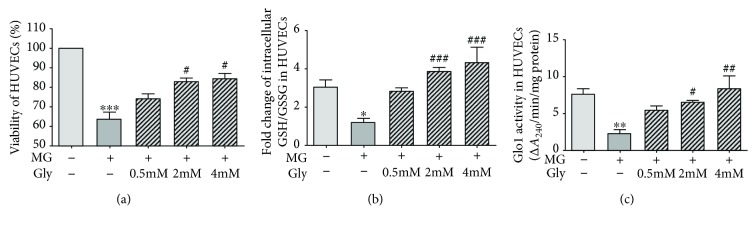
Cell viability and Glo1 function in HUVECs. HUVECs were treated with 400 *μ*M MG and glycine (0.5 mM-4 mM) for 72 h. (a) Cell viability determined by CCK-8. The experiment was performed in triplicate and repeated four times. (b) Intracellular GSH/GSSG ratio in HUVECs. The experiment was performed in triplicate and repeated three times. (c) The activity of Glo1 in HUVECs. The experiment was performed in triplicate and repeated four times. Control: cells incubated in MEM. The medium for all controls and experimental samples had a glucose level of 5.5 mM. MG: methylglyoxal. Gly: glycine. ^∗^*p* < 0.05, ^∗∗^*p* < 0.01, ^∗∗∗^*p* < 0.001 compared with the control group. ^#^*p* < 0.05, ^##^*p* < 0.01, ^###^*p* < 0.001 compared with cells treated with 400 *μ*M MG.

To investigate whether the suppression of AGE formation and the downstream AGE/RAGE signaling pathway caused by glycine treatment was mediated by Glo1, we studied HUVECs. We added 400 *μ*M MG to the HUVEC growth medium to mimic hyperglycemia-induced dicarbonyl stress. During the 72 h incubation with MG, some of the HUVECs were also treated with 0.5 mM, 2 mM, or 4 mM glycine. As shown in Figure 5(a), the cell viability dropped by 35% when treated with MG for 72 h (*p* < 0.001). Both 2 mM and 4 mM glycine protected against the damages caused by MG (*p* < 0.05). The treatment with 0.5 mM glycine had no obvious effect on cell viability.

The incubation of HUVECs with MG caused a significant decrease in the intracellular GSH/GSSG ratio as compared with the control group (*p* < 0.05, Figure 5(b)), but this decrease was reversed by 2 mM and 4 mM glycine treatment (*p* < 0.001).

The incubation of HUVECs with MG also caused a significant decrease in the activity of cellular Glo1 as compared with the control group (*p* < 0.01). As shown in Figure 5(c), the 2 mM and 4 mM glycine treatments significantly elevated the Glo1 activity in a dose-dependent manner (*p* < 0.05 and *p* < 0.01, respectively). No marked alterations in these parameters were detected in cells treated with 0.5 mM glycine.

### 3.8. Glycine Inhibits Cellular AGE Formation and the RAGE-Nox-NF-*κ*B Signaling Pathway in MG-Treated HUVECs

**Figure 6:**
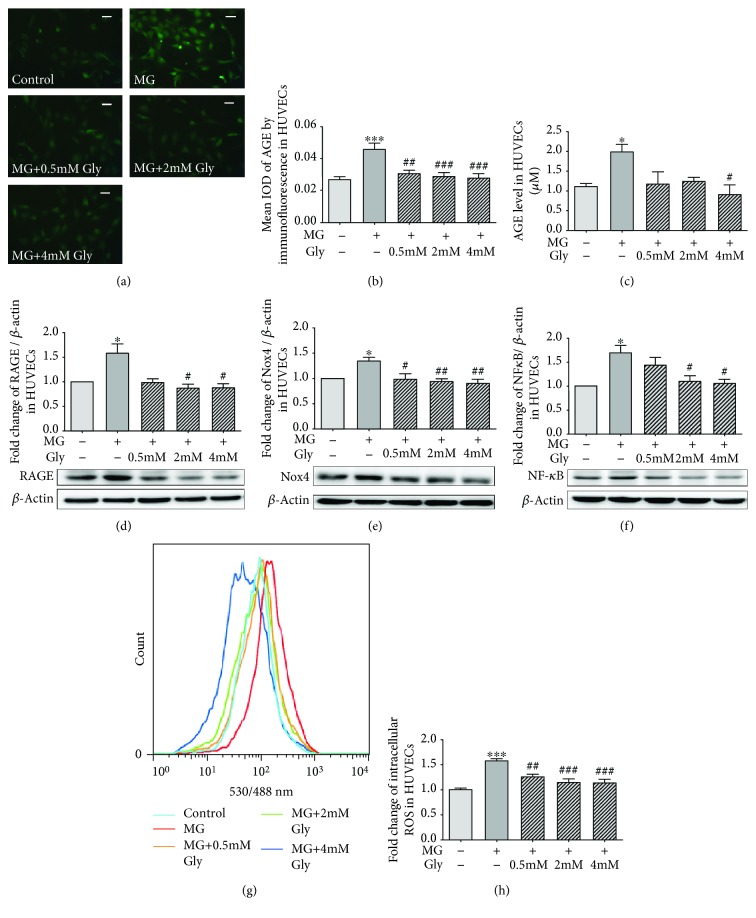
Analysis of the expression of the AGE/RAGE axis in HUVECs. The HUVECs were treated with 400 *μ*M MG and glycine (0.5 mM-4 mM) for 72 h. (a) and (b) show the immunofluorescence analysis of AGE expression levels in HUVECs, magnification 400x. The scale bar indicates 20 *μ*m. The images are the representative of three independent experiments. (c) Intracellular AGE levels determined by ELISA. The experiment was performed in triplicate and repeated three times. The expression levels of RAGE (d), Nox4 (e), and NF-*κ*B p65 (f) in HUVECs were determined by western blot. The experiment was performed in four independent experiments. (g) and (h) show the estimation of intracellular ROS generation by flow cytometry. The images are the representative of four independent experiments. Control: cells treated with 5.5 mM glucose. MG: methylglyoxal. Gly: glycine. ^∗^*p* < 0.05, ^∗∗∗^*p* < 0.001 compared with the control group. ^#^*p* < 0.05, ^##^*p* < 0.01, ^###^*p* < 0.001 compared with cells treated with 400 *μ*M MG.

To investigate whether the favorable effects of glycine on Glo1 function could affect AGE formation and the downstream AGE/RAGE pathway, we determined the levels of AGEs, RAGE, Nox4, and NF-κB p65 in the MG-treated HUVECs. As shown in Figures 6(a) and 6(c), incubation of HUVECs with MG for 72 h significantly increased AGE formation as reflected by immunofluorescence analysis and ELISA (*p* < 0.001 and *p* < 0.05, respectively). The immunofluorescence study showed that AGE expression was decreased by 0.5 mM, 2 mM, or 4 mM glycine treatments (*p* < 0.01, *p* < 0.001, and *p* < 0.001, respectively), whereas the ELISA analysis showed that only the treatment with 4 mM glycine could significantly reverse the increase in AGE formation (*p* < 0.05).

As shown in Figures 6(d) and 6(f), the expression levels of RAGE, Nox4, and NF-*κ*B p65 in HUVECs were significantly increased when treated with MG for 72 h (*p* < 0.05). However, treatment with 2 mM or 4 mM glycine significantly decreased the expression levels of RAGE (*p* < 0.05), Nox4 (*p* < 0.01), and NF-*κ*B p65 (*p* < 0.05). The treatment with 0.5 mM glycine was effective only in suppressing cellular Nox4 expression (*p* < 0.05).

### 3.9. Glycine Treatment Attenuates Intracellular ROS Generation

Flow cytometry analysis showed that after incubating with MG for 72 h, intracellular fluorescence of DCFH-DA, which serves as an approximate estimating tool for intracellular ROS, was significantly increased by 50% as compared with the control group (*p* < 0.001, Figures 6(g) and 6(h)). However, with the treatment with 0.5 mM, 2 mM, or 4 mM glycine, the MG-induced DCFH-DA fluorescence signals were significantly reduced (*p* < 0.01, *p* < 0.001, and *p* < 0.001, respectively).

### 3.10. The Protective Effects of Glycine in Suppressing AGE Formation Are Mediated by Glo1

**Figure 7:**
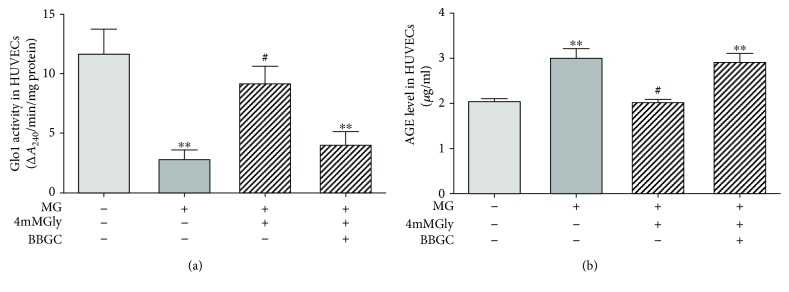
Alterations in Glo1 activity and AGE levels after incubation with the Glo1 inhibitor BBGC for 72 h. Before adding MG and glycine into the medium, the cells in the BBGC group were pretreated with 4 *μ*M BBGC for 12 h. (a) The activity of Glo1 in HUVECs. The experiment was performed in triplicate and repeated four times. (b) Intracellular AGE levels determined by ELISA. The experiment was performed in triplicate and repeated three times. BBGC: S-bromobenzylglutathione cyclopentyl diester. ^∗∗^*p* < 0.01 compared with the control group. ^#^*p* < 0.05, ^##^*p* < 0.01 compared with cells treated with 400 *μ*M MG.

To explore whether the effect of glycine on AGE suppression is mediated by Glo1, we cotreated HUVECs with 4 *μ*M Glo1 inhibitor S-bromobenzylglutathione cyclopentyl diester (BBGC) and glycine for 72 h. Our results showed that the increase in Glo1 activity by glycine was diminished (*p* < 0.01 compared with control, Figure 7(a)). In addition, the suppressive effects of glycine on AGE formation were blocked by BBGC (*p* < 0.01 compared with control, Figure 7(b)).

## 4. Discussion

Oxidative stress plays a central role in the pathogenesis of diabetic vascular complications. Glycine is a precursor of GSH synthesis, and it is known to exert antioxidant effects in models of diabetic complications [17, 19, 25–27]. Studies have shown a role for glycine in restoring vascular endothelial function in aged rats [18] and in rats with metabolic syndrome [13], but the effect of glycine on diabetes-induced macrovascular injuries and the possible mechanisms of any such effect still remain unclear. In the present study, we found that structural impairments in the aorta of diabetic rats, such as distortion of elastic fibers, were markedly ameliorated by glycine treatment. In addition, the decreased levels of NO metabolites in serum and aorta homogenates from diabetic rats were restored by glycine, suggesting that vascular function was improved by glycine treatment. Since oxidative stress can inactivate NO [28] and lead to vascular injury, we therefore hypothesized that glycine may protect vascular tissue by ameliorating oxidative stress.

Although nutritionally nonessential, glycine has been reported to be insufficient in both prediabetic and established diabetic patients [29, 30]. In the present study, we found that the serum levels of glycine in the DM group were decreased compared with those in the control group, whereas the levels of glycine in the DM group were significantly increased as compared with those in the DM group. In normal circumstances, the serum level of glycine in humans varies between 200 and 400 *μ*M [26]. After glycine treatment, the level of glycine in human subjects could even surge to 942 *μ*M, but not causing adverse effects [31]. In our study, the average glycine level in the serum of the healthy rats is 282 ± 6.36 *μ*M, which is about 54% of the level of serum glycine in healthy humans reported by Sekhar et al. (514.7 ± 33.1 *μ*M) [12] but similar to the level of glycine in healthy human subjects reported by Tulipani et al. (272.86 ± 70.78 *μ*M) [29]. Although the concentrations of glycine might differ between rat models and human subjects, substantial studies have shown that in subjects with either diabetes or metabolic syndrome, the levels of glycine are reduced as compared with healthy subjects, but this reduction can be reversed by oral glycine treatment [12, 13, 29, 32].

In parallel with the decreases in glycine levels in the DM group, the GSH levels in the serum and in the aorta homogenates in this group were decreased as compared with those in the control group but were significantly increased in the DG group. Glycine also reduced serum MDA levels and tended to improve serum SOD activity in diabetic rats. Glycine also significantly improved SOD activity and reduced the levels of MDA and 3-nitrotyrosine in aorta homogenates. MDA is an end product of lipid peroxidation caused by ROS generation, and 3-nitrotyrosine serves as a marker of oxidative stress-induced peroxynitrite. Decreases in these oxidative biomarkers in diabetic rats with glycine treatment indicated that the oxidative stress caused by diabetes mellitus was markedly ameliorated by 12 weeks of oral glycine treatment.

It has been reported that AGEs and the downstream AGE/RAGE signaling pathway constitute major mechanisms of vascular oxidative stress [5, 33]. The formation of AGEs is nonenzymatic and irreversible, and many such glycation reactions specifically occur on long-lived macromolecules such as collagen [34], which is found at high concentrations in vessel walls. Once formed, AGEs not only accumulate in the vascular tissue to cause morphological abnormalities but also activate the downstream RAGE-Nox-NF-*κ*B pathway and induce sustained oxidative stress [7, 23]. This hyperglycemia-induced oxidative stress could in turn exacerbate the accumulation of AGEs and the expression of RAGE [1, 16], thus creating a vicious cycle that will lead to constant damage. Therefore, inhibiting AGE formation and oxidative stress is crucial to preventing diabetic vascular complications.

To explore whether glycine ameliorates oxidative stress through the intervention of the AGE/RAGE axis, we assessed the effect of glycine on the expression of AGEs and the downstream AGE/RAGE pathway. The AGE levels in the serum and aorta of diabetic rats were significantly higher as compared with those in the healthy control rats but were significantly lower in the diabetic rats receiving glycine treatment. Meanwhile, the expression levels of RAGE, Nox4, and NF-*κ*B in the aorta of diabetic rats were markedly increased as compared with those in the control group, but this increase was inhibited by glycine treatment. These results demonstrated that glycine treatment significantly inhibited the AGE/RAGE pathway, thus attenuating vascular oxidative stress. Nox4 is a predominant contributor to vascular ROS generation [35, 36]. The decrease in aortic Nox4 expression in the DG group of our study suggested that besides taking part in GSH synthesis, glycine may also work by suppressing the expression of oxidative enzymes. This result was also consistent with our previous study reporting the effects of oral glycine treatment on downregulating islet p22^phox^ expression and oxidative markers in diabetic rat models [37]. In addition, we have recently reported that the 20-week treatment of glycine decreased the levels of mRNA and protein of Nox4 and improved antioxidant defense in the kidneys of STZ-induced diabetic rats [21]. However, in that study, the upstream mechanism whereby glycine regulates renal Nox4 was not investigated. Based on the findings in the present study, it is tempting to speculate that the reduction of Nox4 might result from the suppressive effect of glycine on the expressions of AGEs and RAGE.

The effect of glycine on suppressing aortic AGE formation in our study is of particular interest. To our knowledge, this is the first study that provides evidence that glycine can attenuate AGE formation in vascular tissue. It has been reported that certain bioactive antioxidants, such as ursolic acid [38] and kaempferol [39], can suppress AGE formation and reduce the expression of proteins of the AGE/RAGE axis, thus protecting against oxidative stress and diabetic vascular injuries. Previous studies have reported that glycine might inhibit glucose-induced glycation in the lens by functioning as a scavenger of glucose [27, 40], while another study speculated that this beneficial effect could be attributed to the high solubility of glycine, preventing AGE precipitation [41]. Nevertheless, the molecular mechanisms whereby glycine suppresses AGE formation have not been clearly elucidated.

MG, a highly reactive dicarbonyl compound, has been increasingly recognized as a very important precursor of AGE formation [42]. MG-derived AGEs can bind to RAGE with high affinity and specificity [43], thus activating the AGE/RAGE axis more effectively. In the traditional view, the process of AGE formation is a slow reaction between large biomolecules and reducing sugars such as glucose, but recent studies have noted that MG is up to 200-20,000 times more reactive in AGE formation than glucose [44, 45]. With GSH as a cofactor, Glo1 efficiently detoxifies MG, thus preventing AGE formation. GSH is a critical antioxidant in the human body, but the level of GSH is insufficient in diabetic patients due to excessive oxidative stress [12, 46]. Additionally, it has been reported that the activity of Glo1 *in vivo* is proportional to the cellular concentration of GSH [11, 14]. The fact that glycine increases GSH synthesis and suppresses AGE formation in our study led us to the hypothesis that glycine might exert beneficial effects on Glo1 function, thus protecting against AGE formation. Therefore, we measured the expression and activity of aortic Glo1. Our results showed that Glo1 expression and activity in the aorta were reduced in the DM group as compared with the control group, indicating that the ability of Glo1 to degrade MG was impaired. The decreased Glo1 function also partly accounted for the increase in AGE formation in the diabetic rats. Compared with those in the DM group, the expression and activity of aortic Glo1 in the DG group were significantly increased. Therefore, it is reasonable to speculate that glycine may restore Glo1 function by increasing GSH levels and improving Glo1 expression and activity, thus inhibiting AGE formation and subsequent oxidative stress.

To confirm that the suppressive effect of glycine on the AGE/RAGE axis was associated with the observed improvement in Glo1 function, we incubated the HUVECs directly with 400 *μ*M MG for 72 h to mimic the dicarbonyl stress induced by hyperglycemia. Our results showed that in MG-treated HUVECs, the administration of 4 mM glycine significantly increased the activity of Glo1 as well as the intracellular GSH/GSSG ratio, indicating that the function of Glo1 was restored by glycine treatment. Glycine administration also resulted in a reduction in the formation of cellular AGEs and in significantly decreased expression levels of several AGE/RAGE downstream signaling pathway proteins, including RAGE, Nox4, and NF-*κ*B p65. Consequently, MG-induced intracellular oxidative stress was significantly attenuated by glycine administration. In addition, we found that the Glo1 inhibitor BBGC significantly blocked the suppressive effects of glycine on AGE formation. Thus, our in vitro experiment provided evidence of the beneficial effects of glycine on preventing MG-induced activation of the AGE/RAGE axis and subsequent increases in oxidative stress.

Endothelial cell injury is an early event in the development of atherosclerosis. Loss of intracellular glutathione is associated with disturbances in endothelial barrier function [47]. The equilibrium between the reduced form of glutathione (GSH) and the oxidized form (GSSG) is crucial for the maintenance of cellular redox status. When GSH is oxidized to scavenge free radicals, the oxidized glutathione (GSSG) can be recycled to regenerate GSH by a pathway that uses NADPH as a cofactor. However, under chronic oxidative stress, NADPH is consumed by the polyol pathway, thus impairing glutathione recycling [1]. In our study, the oxidative stress caused by MG treatment markedly depleted the reduced form of glutathione (GSH) and increased GSSG content in the HUVECs, thus decreasing the GSH/GSSG ratio and consequently impairing Glo1 function. When intracellular GSSG approaches cytotoxic levels, it might be transported outside the cells [48], which may eventually lead to an insufficiency of total glutathione content due to the unavailability of substrates. This hypothesis is supported by the significant decrease in total glutathione levels in the serum and in the aortic tissue homogenates from the diabetic rats in our study. However, in the DG group, both the total glutathione content *in vivo* and the GSH/GSSG ratio *in vitro* were significantly increased as compared with those in the DM group. This effect of glycine supplementation on maintaining the GSH equilibrium is supported by other studies that have reported that oral glycine treatment restores impaired GSH levels in diabetic or metabolic syndrome models [12, 13, 37]. Additionally, glycine might even be the rate-limiting factor in GSH synthesis in the tissue, according to a study by Mohammed et al. [32] which emphasized the role of glycine in maintaining antioxidant defense.

Considerable evidence has shown that the expression of Glo1 can be upregulated by bioactive antioxidants [49–52]. Although the mechanisms still remain unclear, the role of the nuclear translocation of nuclear factor erythroid-2-related factor 2 (Nrf2) has been increasingly acknowledged. Nrf2 is a redox-sensitive transcription factor that regulates the expression levels of various antioxidant enzymes, including SOD, heme oxygenase 1, and glutamylcysteine synthetase. It was recently reported that Nrf2 also binds to the antioxidant response elements (ARE) in exon 1 of Glo1 [53]. Therefore, translocation of Nrf2 into the nucleus could increase the expression of Glo1 and enhance its activity. We have previously found that glycine could promote nuclear translocation of Nrf2 in the kidney of diabetic rats (unpublished data), but the effect of glycine on Nrf2 activation in the vascular tissue has not been investigated. Nevertheless, this leaves open the possibility that the beneficial effects of glycine on vascular Glo1 function might be attributed to Nrf2 nuclear translocation.

This study indeed has some limitations. The MG levels in different groups were not measured in the animal experiment; thus, the direct function of Glo1 in degrading MG was not determined. Therefore, in the *in vitr*o experiment, we directly incubated HUVECs with MG to investigate the effects of glycine on MG-induced damage in vascular cells and confirmed that glycine was able to protect Glo1 function. Moreover, in our study, the concentrations of glycine used in the cell culture experiments were higher than the levels of glycine measured in animal serum. In the HUVECs, we started with 0.5 mM glycine, because this concentration was close to the average serum glycine levels detected in the DG group of the animal experiment. However, 0.5 mM glycine was not sufficient to protect against MG-induced cell damage. Therefore, the glycine concentration was increased to 2 mM and 4 mM. In addition, in the cell experiments, we only used the Glo1 inhibitor to interfere with the function of Glo1, which might result in nonspecific effects. The knockdown of Glo1 using siRNA would provide a complement to the inhibitor study and better demonstrate the specific mechanism of the effect of glycine on Glo1.

In summary, our study demonstrated that glycine attenuates oxidative stress in the aorta of diabetic rats by inhibiting AGE accumulation and the subsequent RAGE-Nox-NF-*κ*B signaling pathway. In addition, the beneficial effect of glycine on suppressing AGE formation may be associated with increasing Glo1 activity and GSH synthesis. The precise mechanism underlying the role of glycine in protecting against diabetic macrovascular injuries still needs further investigation, in an effort to expand the treatment options available for clinical practice.

## Figures and Tables

**Table 1:** Plasma glucose levels, body weight, and serum glycine levels after 12 weeks of treatment.

	Control	CG	DM	DG
Plasma glucose (mmol/l)	6.03 ± 0.64	5.7 ± 0.77	24.07±2.09^∗∗∗^	20.80±2.57^∗∗∗^
Body weight (g)	643.5 ± 6.56	647.8 ± 8.93	454.6±7.52^∗∗∗^	452.2±8.54^∗∗∗^
Serum glycine (*μ*M)	282 ± 6.36	445.43±8.63^∗∗∗^	217.15 ± 5.86^∗^	430.71 ± 8.39^###^

Control: healthy rats receiving normal tap water. CG: healthy rats receiving tap water with 1% glycine added. DM: diabetic rats receiving normal tap water. DG: diabetic rats receiving tap water with 1% glycine added. *N* = 7-8. ^∗^*p* < 0.05, ^∗∗∗^*p* < 0.001 compared with the control group. ^###^*p* < 0.001 compared with the DM group.

## Data Availability

The data used to support the findings of this study are available from the corresponding author upon request.
